# An Observational Prospective Clinical Study for the Evaluation of a Collagen-Hydroxyapatite Composite Scaffold in Hip Revision Surgery

**DOI:** 10.3390/jcm11216372

**Published:** 2022-10-28

**Authors:** Pietro Cimatti, Isabella Andreoli, Maurizio Busacca, Marco Govoni, Leonardo Vivarelli, Nicolandrea Del Piccolo, Alessandra Maso, Cesare Stagni, Giovanni Pignatti

**Affiliations:** 1 Reconstructive Orthopaedic Surgery and Innovative Techniques—Musculoskeletal Tissue Bank, IRCCS Istituto Ortopedico Rizzoli, 40136 Bologna, Italy; 2 Montecatone Institute Rehabilitation Hospital, 40026 Imola, Italy; 3 Laboratory of Microbiology and GMP Quality Control, IRCCS Istituto Ortopedico Rizzoli, 40136 Bologna, Italy; 4 Rizzoli Sicilia Department, IRCCS Istituto Ortopedico Rizzoli, 90011 Bagheria, Italy

**Keywords:** hip revision surgery, bone tissue regeneration, collagen-based hydroxyapatite, stemmed cup

## Abstract

One of the greatest challenges of hip revision surgery is the need to restore extensive bone loss by creating a stable reconstruction with long-term durability. The present observational, investigator-initiated prospective study was carried out to evaluate the clinical and radiological results of the use of a commercial biomimetic collagen–hydroxyapatite composite biomaterial (RegenOss) applied in hip revision surgery. Thirty-three patients who underwent hip revision were included in this study, and 29 received up to 2 years of follow-up. The acetabulum was reconstructed using an uncemented hemispherical shell both with or without an iliac fixation stem. Functional recovery was assessed according to the Harris Hip Score (HHS) at the pre-hospitalisation check-up, and at 6-, 12-, and 24-month follow-ups. Radiological evaluation consisting of X-ray analyses (6, 12, and 24 month follow-ups) and CT scan exams (within 10 weeks post-surgery and at 12-month follow-up) were performed to evaluate the reduction in bone defect and new bone regeneration. All the patients reported a complete recovery and a considerable improvement in functional outcome assessed by the HHS, which was significantly higher at all the follow-ups than at pre-hospitalisation. Moreover, radiological assessments revealed good scaffold integration. Overall, collected data suggest that RegenOss is a valid and safe alternative to restoring acetabular bone loss in revision hip arthroplasty.

## 1. Introduction

With the ageing population and the increase in life expectancy characteristic of the Western World, especially in recent years, there has been a greater demand for total hip replacement procedures [1], followed by an increase in hip revision surgeries. Stability and long-term fixation of implants in revision hip arthroplasty are determined by many factors, such as bone tissue availability, adequate implant geometry, and stable fixation. Based on the extent of the bone defect, there is a wide variety of orthopaedic implants available, including cemented or uncemented cups, porous implants with iliac fixation stem, and reinforcement devices such as cage, ring, plate, or mesh [2,3,4,5,6,7,8,9,10].

Over the years, several material options have been investigated for bone repair and regeneration [3,4]. However, to date, autologous bone is still considered the “gold standard” for graft procedures due to its osteoconductive, osteoinductive, and osteogenic properties. Nevertheless, many factors, (e.g., limited availability, donor site morbidity, possible vascular and neurological complications, the increase in surgery times, blood loss, and postoperative pain) limit the harvesting and use of autogenous bone tissues [11,12].

Allografts have been widely used to treat bone loss, yet not without several drawbacks concerning the processing, preservation, and storage, which may affect the mechanical properties of the tissue and increase the risk of implant migration and loosening [13,14]. However, it should be noted that allografts are procured, processed, and distributed only by tissue banks, operating under strict guidelines and in sterile conditions, minimising the abovementioned issues [15].

Nevertheless, to overcome the limitations of these treatments, especially those related to the lack of availability of patient/donor tissue, synthetic or semi-synthetic materials have been demonstrated to be valid alternatives for the replacement of natural bone stock [16,17,18,19,20,21]. Among these, absorbable ceramics such as hydroxyapatite (HA), calcium sulphate, and tricalcium sulphate (TCP), or their combination, are commonly used as bone grafts [22]. Notably, HA is a major component of natural bone and can favour new bone formation while acting as a scaffold in the implanted site. Several preclinical and clinical studies have demonstrated that hydroxyapatite-based bone substitutes represent a safe and effective solution for bone regeneration [12,23,24,25]. Throughout the years, extensive research efforts have been dedicated to the development of hydroxyapatite-based scaffolds [26] with improved properties in terms of biomimetics, osteointegration, resorption kinetics, and bone regeneration. In this regard, multi-substituted non-stoichiometric hydroxyapatites and calcium phosphates combined with natural proteins (e.g., collagen, alginate, chitosan), have shown an improvement in the reabsorption properties and performances in tissue regeneration [27,28,29,30,31].

Among collagen-HA-based materials, RegenOss (Finceramica S.p.A., Faenza, Italy) is a three-dimensional composite biomimetic scaffold characterised by magnesium-enriched hydroxyapatite nanocrystals, nucleated into type I collagen fibres. RegenOss has previously shown its bone regenerative properties and safety profile, both in vitro [32,33,34,35] and in vivo preclinical [36,37] and clinical studies [38,39,40,41,42]. Therefore, driven by these encouraging published results, the present observational prospective study used RegenOss with different cup types in hip-revision procedures to evaluate the clinical and radiological outcomes up to 2 years post-surgery.

## 2. Patients and Methods

### 2.1. Study Design and Setting

This was an observational prospective clinical study conducted following the Declaration of Helsinki at the IRCCS Istituto Ortopedico Rizzoli (Bologna, Italy). The protocol was approved by the Ethics Committee of the same institute on 3 July 2012 (REVISION_0022989). All subjects signed the informed consent for inclusion before they participated in the study.

### 2.2. Patients

Between July 2012 and May 2014, 33 patients requiring hip revision were enrolled in the study. As reported in Table 1, the patient population was composed of 21 females and 12 males (ratio 1.75 F/M) with an average age of 64.5 ± 10.6 (age range: 43 to 79 years old) at the time of surgery. Mean height, weight, and body mass index (BMI) were 163 cm (range: 142–182 cm), 72 kg (range: 53–97 kg), and 27.2 ± 3.7 kg/m^2^ (range: 20–38), respectively. 

Fifteen patients presented a lesion on the left hip, while 18 presented a lesion on the right hip. Acetabular defects were mainly due to degenerative joint diseases (i.e., arthrosis; *n* = 26, 6 of which presented arthrosis secondary to congenital hip dysplasia), traumatic events (*n* = 3), or osteoarthritis (*n* = 4). The causes of defects were mainly due to mechanical acetabular loosening (*n* = 13), mechanical total loosening (*n* = 8), implant instability/dislocation (*n* = 8), and septic loosening (*n* = 4) before first revision surgery.

In patients where instability/dislocation prosthetic issues have led to the replacement of an existing prosthesis, as well as for patients with mechanical acetabular/total loosening, the surgical intervention involved removing the previous cup and inserting a new one with the addition of RegenOss to fill the bone defect which occurred during the cup removal. On the other hand, in case of bone loss caused by an infection, the prosthetic replacement was carried out in two stages: In the first stage, the mobilised and infected primary prosthesis was removed and replaced with a cemented spacer. In the second stage, after antibiotic therapy, normalisation of phlogosis markers, and negative bone scintigraphy, a new prosthesis was placed together with RegenOss to fill the bone gap.

According to the Paprosky classification [43], which is a categorisation based on bone loss location and degree of severity (grades: I, IIA, IIB, IIC, IIIA, IIIB, IV), 26 hips were classified as type IIB, 2 hips were type IIC, 2 hips were type IIIA, and 3 hips were classified as IIIB.

Nineteen (57.6%) subjects underwent their first hip revision surgery, 9 (27.3%) underwent a second hip revision, and 5 (15.1%) underwent a third hip revision surgery. Twenty-one patients (63.6%) underwent head and acetabular revision, and the remaining 12 (36.4%) were operated on for total hip revision.

Materials coupling in implanted prosthesis and the relative number of patients are listed as follows: ceramic-on-ceramic (20 patients); polyethylene-on-ceramic (4 patients); polyethylene-on-metal (8 patients); and polyethylene-on-polyethylene (a single case). A Sansone iliac stem cup was used in 8 subjects (24.2%), while a press-fit HA-coated cup without an iliac fixation stem was used in the remaining 25 patients (75.8%).

### 2.3. Inclusion and Exclusion Criteria

To obtain a reliable assessment of the reconstructed outcomes, patients were selected based on the following criteria:

- The Paprosky classification ranging from type IIB to IIIB;
- Standard clinical practice in hip prosthesis revision surgery with associated bone loss;
- Limited bone defect;
- Uncemented cups (HA-coated).

On the other hand, patients were excluded for the following criteria:

- Age < 18;
- Lacking written informed consent;
- Second revision surgery was required due to previous or existing infection;
- Patients with diseases affecting bone quality, such as osteoporosis, rickets, osteomalacia, and Paget’s disease;
- Patients with comorbidities, such as uncontrolled high blood pressure, coronary heart disease, cerebrovascular disease, diabetes or other endocrine diseases, tumours, inflammations, metabolic diseases, presence of imbalance of nerves and muscles that could affect the lower limb function, and other conditions that may affect the outcomes assessment.

### 2.4. Outcome Collection

Basic information, including demographic characteristics and clinical information, was collected for all patients, as previously reported in Table 1. Moreover, as shown in Table 2, during the pre-admission visit, the Harris Hip Score (HHS) was collected. This is used to evaluate various hip disabilities such as pain, impaired physical function, and range of motion in an adult population. HHS scores range from 0 to 100 (higher scores representing less dysfunction and better outcomes). HHS was also collected during the follow-ups scheduled at 6, 12, and 24 months after the surgery.

Radiological evaluation was carried out during the pre-admission visit, and at the 6-, 12-, and 24-month follow-ups. Some X-ray scales used to examine bone healing could minimise the heterogeneity of assessment methods [44,45,46]. In this respect, the features measured and analysed from these radiographs were radiolucent lines, position and migration of the acetabular prosthetic components, and incorporation of the bone substitute.

Radiographs taken in the immediate postoperative period and at the most recent follow-up were evaluated considering the presence of radiolucent lines around the acetabular components according to the three zones defined by DeLee and Charnley [44]. Radiolucency greater than 1 mm in width in any portion of a zone was considered significant. An acetabular component was considered loose when a continuous radiolucent line of more than 1 mm in width was present around the entire porous-coated region of the component. The measurement of migration of the acetabular component and the preoperative and postoperative centres of hip rotation were estimated by measuring the position of the implant from the fixed pelvic landmarks. A linear change greater than 3 mm or a rotational change greater than 5° was a sign of component migration.

Finally, bone graft material was considered incorporated when the radiographs showed evidence of trabecular bridging between the graft and host bone.

Computed tomography (CT) exams were conducted between 5 and 10 weeks, namely when osseointegration had not yet occurred, and were performed at the 12-month follow-up on 14 of the 33 enrolled patients (precisely 14.4 ± 2.9 months; time variation depended on patient availability). Nine patients did not show up for the scheduled exams, 3 withdrew from the study during follow-ups, and 1 passed away. Lastly, although another 6 patients underwent CT scans, data were not taken into account since the exams were performed outside of the follow-up range.

The CT volumetric acquisitions, performed with a 64-slice VCT LightSpeed^®^ scanner (GE Healthcare, Milwaukee, WI, USA), were conducted from the supratectal region to the lower limit of the prosthetic stem, with a 1 mm thickness and 1 mm interval, using bone and soft tissue reconstruction algorithms. From the “basal” axial images, post-processing reconstructions on the sagittal plane were obtained and used as a reference to select a series of slices on the oblique axial plane, with a 0.7 mm thickness and an interval of 3 mm, parallel to the base of the cup.

A series of oblique coronal reconstructions conducted perpendicularly to the acetabular cup was also obtained, using basal axial images as a reference.

From the abovementioned series, all the slices included between the upper and the lower limits of the acetabular cup were selected for the axial plane (from 12 to 16 images), while for the reconstructions obtained on the oblique coronal plane, the selection included all the images between the posterior and the anterior edges of the acetabular cup (from 11 to 15 images).

Three expert radiologists in the field of musculoskeletal apparatus measured the area (mm^2^) of the defect in all reconstructed axial and coronal oblique images on the high-resolution monitors of the Picture Archiving and Communication System (PACS; Carestream Health Inc., Rochester, NY, USA). The measurements obtained by each radiologist were included in the medical report of the two exams, keeping them separate from the two CT controls and the two different planes, obtaining an average for each plane and exam. The average of the values obtained by the three radiologists in the axial oblique plane and the coronal oblique plane was then calculated. The overall averages obtained in the first examination and in the 1-year follow-up examination were then compared to quantify the extent of bone regeneration and the possible reduction of the defect.

Finally, all adverse events were recorded by the surgeon in the case report form (CRF).

### 2.5. Medical Device Description

#### 2.5.1. Bone Graft Substitute

All the patients received RegenOss bone graft substitute. RegenOss is an implantable, resorbable, bioactive bone substitute for bone defect reconstruction. The composite device is flexible and is claimed to reproduce the anatomical structure of natural bone tissue. RegenOss consists of type-I collagen (equine source) and hydroxyapatite enriched with magnesium ions. Collagen is a fibrous protein and represents the most important structural component of the extracellular matrix of many human connective tissues, while hydroxyapatite is the main bone mineral element. Therefore, the presence of type-I collagen (i) makes this device chemically and structurally biomimetic, (ii) ensures in-site stability, and (iii) can favour bone tissue regeneration processes.

#### 2.5.2. Prosthesis

Thirty-three retrieved cups were all solidly fixed without loosening. Twenty-nine patients had one-stage revision surgery, while 4 had a two-stage revision. FIXA Ti-Por (Adler Ortho^®^ S.p.A., Cormano, Italy), HERM-BS-Sansone (Citieffe s.r.l., Calderara di Reno, Italy), and Tritanium (Stryker Orthopaedics, Mahwah, NJ, USA) acetabular cups were used in 20 (60.6%), 8 (24.2%), and 5 (15.2%) patients, respectively.

### 2.6. Surgical Procedure

All procedures were performed by four expert orthopaedic surgeons using a lateral approach.

Satisfactory intraoperative stability and range of motion (ROM) were achieved in all cases without the need for augments, including in type III defects in which generally a stable fixation of the prosthesis is requested to achieve a proper stabilisation (examples are provided in the Supplementary Materials, such as Figure S1).

### 2.7. Statistical Analysis

Summary statistics were appropriately presented (mean, standard deviation, median, minimum and maximum for continuous variables, and absolute and relative frequencies for discrete variables) and 95% confidence intervals were computed for continuous variables. One sample *t*-test was performed to evaluate the statistical significance of the differences between pre-operative and post-operative HHS and radiological values. Differences were considered statistically significant when *p* < 0.05. All statistical analysis was performed with SAS Statistical Software, version 9.4 (SAS Institute Inc., Cary, NC, USA).

## 3. Results

Between July 2012 and May 2014, 33 patients requiring hip revision were enrolled in the study.

The follow-up was carried out at 6, 12, and 24 months. However, during the 6-month follow-up, one patient passed away due to causes neither related to the surgery nor the applied devices, while one patient withdrew during the follow-up. Among the 31 remaining patients, two subjects also withdrew during follow-up within 12 months after surgery. The classification of patient populations was therefore considered as follows: (I) the clinical population, composed of all the patients who had evaluable clinical results by HHS at 2 years follow-up (*n* = 29); (II) the CT population, composed of all patients who had a post-operative CT scan evaluation within 10 weeks after the surgery and at the 12-month follow-up (*n* = 14); and (III) the enrolled population, composed of all the patients treated for hip revision and receiving the graft substitute RegenOss (*n* = 33).

### 3.1. Clinical Results

Clinical evaluation was performed on a total of 29 patients who participated in all follow-ups up to 24 months. HHS values collected at pre-surgery, and at 6, 12, and 24 months were 46.7 ± 17.52, 81.6 ± 10.79, 84.7 ± 11.78, and 87.2 ± 11.60, respectively. As shown in Table 3, the comparison of the pre-surgery HHS with 6-, 12-, and 24-month HHS values revealed a statistically significant (*p* < 0.001) improvement during all follow-ups.

Likewise, the differences between pre-operative and 6-, 12-, and 24-month post-operative HHS were all statistically significant (*p* < 0.001), both in the group of first revisions (Table 4) and in the groups of second and third revisions (Table 5).

Seventeen of the 29 patients underwent their first acetabular cup revision, while 12 underwent a second (*n* = 7) or a third (*n* = 5) revision.

In the first revision group, HHS values collected at pre-surgery, and at 6, 12, and 24 months were 48.6 ± 15.46, 85.4 ± 9.69, 89.9 ± 7.81, and 91.9 ± 8.03, respectively.

In the second and third revision group, HHS values collected at pre-surgery, and at 6, 12, and 24 months were 44.1 ± 20.30, 76.1 ± 10.20, 77.2 ± 12.66, and 80.5 ± 12.87, respectively.

Several clinical subgroups were identified for clinical outcomes evaluation, as reported in Table 6, Table 7, Table 8 and Table 9. For each subgroup, the difference between the pre-operative and 24-month post-operative HHS values were all statistically significant. However, no statistical evaluation was performed among the different subgroups, because of the low number of patients within each subgroup.

### 3.2. Radiological Results

**Figure 1:**
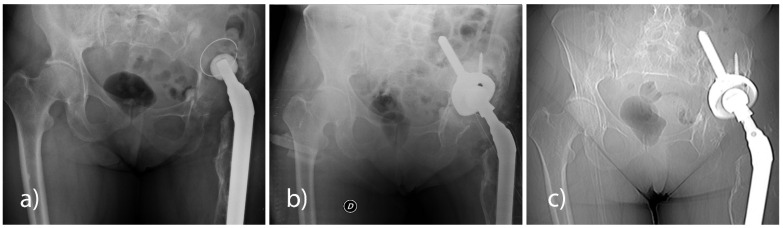
(**a**) Aseptic mobilization of the right hip prosthesis cup. (**b**) 6-month post-operative radiographic control shows the revised cup (Sansone cup with an iliac stem and RegenOss) stably in place. (**c**) A 12-month CT scan shows no signs of prosthetic loosening.

All patients underwent a pelvis X-ray check-up in AP projection in a standing position and with a lateral hip view at pre-hospitalisation and at 6, 12, and 24 months post-surgery. None of the patients had any signs of loosening of the implanted cup (Figure 1).

Furthermore, 14 patients also underwent CT evaluation within 10 weeks from surgery and at the 12-month follow-up after surgery.

**Figure 2:**
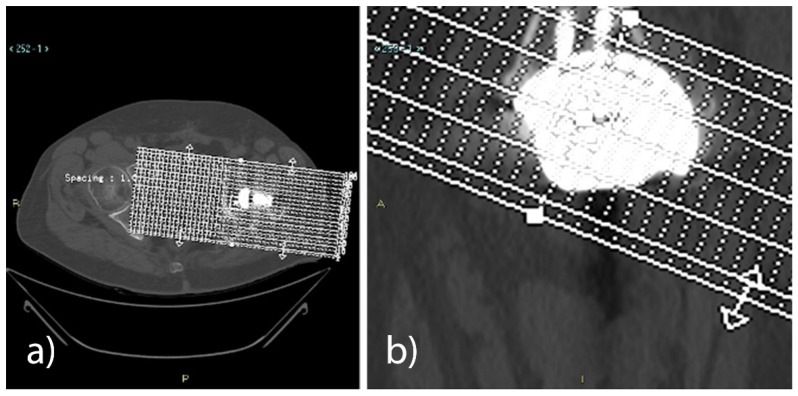
(**a**) Axial and (**b**) coronal CT views for bone gap measurement.

Compared to the first CT exam (5–10 weeks), the 12-month CT follow-up in all the analysed patients revealed a statistically significant reduction (*p* < 0.001) of the bone gap, both in the axial plane (23%) and in the coronal plane (21%). With the use of either the Sansone iliac stem cup or the press-fit cup without an iliac stem, a reduction of the bone gap was revealed in the axial plane and the coronal plane at the 12-month CT follow-up (Table 10 and Table 11, Figure 2).

### 3.3. Adverse Events

Three patients reported adverse events in the first 6 postoperative weeks, all related to the surgery. One patient showed mechanical complications (i.e., prosthetic dislocation) and infection (i.e., secreting fistula); the event was resolved by surgical cleaning of the implanted site and brace application. One patient presented a superficial infection of the surgical wound at a 6-week post-operative check-up; the event was resolved by surgical cleaning. One patient died of a heart attack within 6 months of follow-up. This event was considered by the investigator to be related neither to the device nor to the surgery.

No cases of deep infection or mechanical instability were related to RegenOss. No cases of allergic reactions were recorded. The survival rate of the bone substitute at a 2-year follow-up was 100%.

## 4. Discussion

Revision surgery is often more complex and time-consuming than primary joint replacement. Patients are frequently asymptomatic for years before presenting to the orthopaedic surgeon. Nevertheless, when symptoms occur, it is frequently found that the components have loosened, leading to large defects in the original bone. The patient’s bone stock is frequently of poorer quality than at the primary operation and, considering the concurrent difficulties in removing the old metallic implant, the orthopaedic surgeon must address substantial patient bone defects [47].

Autologous bone remains the “gold standard” due to its unsurpassed biological activity, both in the reconstruction of bone defects and in implant sites with low osteogenic potential. The main disadvantages of human autografts are their limited quantity and the morbidity associated with their harvest. Human allografts offer an abundantly available alternative, which can circumvent the potential morbidity of autograft harvesting. However, their use relies on sophisticated bone banking systems, which work under stringent guidelines to avoid potential disease transmission or other issues mainly associated with tissue collection, processing, preservation, and storage. Therefore, based on the biomimetic principle, although a material as similar as possible to the host bone is recommended to allow the best biological behaviour [48], relative concerns over the use of autografts or allografts have led to the development of numerous synthetic bone graft substitutes. In this regard, several commercial bone substitutes have been demonstrated to restore bone loss and withstand the dynamics of compaction [12], while having advantages such as an unlimited supply, easy sterilisation, and room temperature storage [49].

Among synthetic bone substitutes, the use of bioceramics in procedures of hip revision as graft extenders for acetabular bone loss has been documented for years with satisfactory results [16,25,47,50]. Mixed with human bone or other natural materials, or combined with different types of cups, bioceramics have shown their safety profile at long-term follow-up.

It is well-known that collagen is a fibrous protein which represents the most important structural component of the extracellular matrix of many human connective tissues. Therefore, adding type-I collagen to the formulation of bone substitutes means improving the chemical–structural biomimetic properties of the product, ensuring in-site stability, and favouring bone tissue regeneration and repair.

RegenOss is a composite bone substitute which follows the principles of self-healing guided tissue regeneration by biomimetics. The removal of the damaged tissue creates a bone gap guaranteeing adequate blood flow. RegenOss is highly hydrophilic and can be adapted to the gap to create an anatomic continuity. Therefore, once implanted, it essentially acts as a temporary template for host cell adhesion and proliferation, without causing significant inflammatory or adverse reactions, favouring self-healing and tissue repair.

In the present observational clinical study, the claimed performance of RegenOss was evaluated in terms of bone tissue replacement and functional recovery. The achievement of success regarding these conditions was expected to allow for an improvement in the patient’s general health.

The device was used alone and mixed with the patient’s blood to recover the bone loss. Specifically, bone tissue regeneration was evaluated using a CT scan, a technique that allows a comprehensive assessment of hip replacements (implant components, bone and/or cement interfaces, bone stock, and soft tissue). However, evaluating the bone defect in prosthetic re-implants and any regenerated bone is generally very difficult with imaging methodology. For instance, it is not possible to use magnetic resonance imaging (MRI) due to the artefacts caused by the metal components of most prostheses on the market. Likewise, CT scans also have numerous artefacts that hinder the interpretation of the exam; however, our measurement method allowed us to retrieve reliable and reproducible CT measurements between different exams.

The performance of RegenOss was confirmed by the evidence of good host-integration of the bone substitute and bone regeneration at the site of implant. This was shown using radiological imaging, which highlighted a reduction of bone gap and new bone formation in all observed patients. The device proved to be an effective treatment option to restore acetabular bone stock in first, second, and third hip revision surgery, also with an advanced bone loss (type IIIA–IIIB according to Paprosky’s classification). Functional recovery was confirmed by statistically significant improvements in the Harris Hip Score up to a 2-year follow-up, compared to pre-operative values. Furthermore, the safety of the device was confirmed during the long follow-up period, as none of the treated patients developed post-op deep infections or inflammatory reactions due to the material.

The main limitations of the present study were the small sample size and the limited number of patients who underwent CT scan evaluations during follow-ups. Other limitations are related to the observational study design such as the lack of randomisation, which makes observational studies confounding and more prone to bias. Moreover, it is worth noting that heterogeneity of the patient population, such as different (i) pre-operative diagnoses, (ii) types of defects, and (iii) types of implants, may reduce the strength of the study and affect the results of bone healing. In this respect, to avoid potential bias and/or misleading results related to the abovementioned issues, we divided the studied population into clinical subgroups, as reported in Table 6 and Table 9, where patients were evaluated in terms of different prosthesis materials coupling and cup type. For each subgroup, the difference between the pre-operative and 24-month post-operative HHS values were all statistically significant. Likewise, CT scan evaluation showed that axial and coronal plane changes between 5 to 10 weeks and 12 months were statistically significant both for patients treated with the Sansone iliac screw cup and the press-fit cup without iliac screw, as respectively reported in Table 10 and Table 11.

Therefore, taken together, reported results on the use of RegenOss in hip revision surgery in terms of expected safety and performance were considered satisfactory. Additionally, serious postoperative complications related to the graft material did not occur.

## Figures and Tables

**Table 1:** Patient population: demography, aetiology, classification of defects, and information on surgery.

Demography	Gender	Male	Female
	No. of patients	12	21
		Mean ± SD	Min–max
	Age at surgery	64.5 ± 10.6	43–79
	BMI ^1^ kg/m^2^	27.2 ± 3.72	20–38
Aetiology			Count
	Lesion site	Left	15
		Right	18
	Lesion origin	Traumatic events	3
		Osteoarthritis	4
		Degenerative joint disease (arthrosis)	26
	Lesion cause	Instability/dislocation/failure	8
		Mechanical acetabular loosening	13
		Septic loosening (joint infection)	4
		Mechanical total loosening	8
Defect classification	Paprosky classification	No. of patients	Mean age
	IIB	26	66
	IIC	2	60
	IIIA	2	67
	IIIB	3	55
Surgery			Count
	Revision	1st	19
		2nd	9
		3rd	5
	Revision type	Total	12
		Partial (head + acetabular)	21
Materials			Count
	Coupling type	Ceramic-on-Ceramic	20
		Polyethylene-on-Ceramic	4
		Polyethylene-on-Metal	8
		Polyethylene-on-Polyethylene	1
	Cup type	Sansone iliac screw cup	8
		HA-coated cup without iliac screw	25

^1^ BMI: body mass index.

**Table 2:** Schedule of pre-hospitalisation, intervention, and assessments in the study.

	Pre-Admission Visit	Surgery	Follow-Up			
			5–10 Weeks	6 Months	12 Months	24 Months
Inclusion/Exclusion Criteria evaluation	×					
Signed Informed consent form collection	×					
Demographic data collection	×					
Surgical report ^1^		×				
Total Harris Hip Score	×			×	×	×
X-Ray evaluation	×			×	×	×
CT scan evaluation			×		×	

^1^ Information included in the surgical report: material, prosthesis, associated surgery, diagnostic examination, type of revision, and support associated with prosthesis.

**Table 3:** Mean HHS values of all revisions in the different follow-up intervals and their comparison with the pre-surgical mean value.

Harris Hip Score	No.	Mean	SD	Median	Min	Max	CI 95%	*p*
Variation: pre-s./6 m.	29	35.2	17.29	37	3	70	28.7–41.8	<0.001
Variation: pre-s./12 m.	29	36.0	19.13	40	1	72	28.8–43.3	<0.001
Variation: pre-s./24 m.	29	38.6	18.45	40	2	72	31.5–45.6	<0.001

**Table 4:** Mean HHS values of first revisions in the different follow-up intervals and their comparison with the pre-surgical mean value.

Harris Hip Score	No.	Mean	SD	Median	Min	Max	CI 95%	*p*
Variation: pre-s./6 m.	17	37.6	15.43	35	9	60	29.7–45.6	<0.001
Variation: pre-s./12 m.	17	40.4	16.27	41	15	64	32.0–48.8	<0.001
Variation: pre-s./24 m.	17	42.4	16.20	41	17	70	34.0–50.7	<0.001

**Table 5:** Mean HHS values of second and third revisions in the different follow-up intervals and their comparison with the pre-surgical mean value.

Harris Hip Score	No.	Mean	SD	Median	Min	Max	CI 95%	*p*
Variation: pre-s./6 m.	12	31.8	19.83	37	3	70	19.2–44.4	<0.001
Variation: pre-s./12 m.	12	29.8	21.80	31.5	1	72	16.0–43.7	<0.001
Variation: pre-s./24 m.	12	33.2	20.76	38.5	2	72	20.0–46.4	<0.001

**Table 6:** Total Harris Hip Score (HHS) pre-op vs 24 months’ follow-up values in subgroups of prosthesis materials coupling type.

Prosthesis Materials Coupling	*n*. of Patients	Total HHS Value			*p*
		Pre-op Mean	24 Months Follow-Up Mean	Variation Mean	
Ceramic-on-Ceramic	17	49.8	86.8	37.0 (CI: 28.4%–45.6%)	<0.001
Other	12	46.9	87.7	40.8 (CI: 27.3%–54.2%)	<0.001

**Table 7:** Total HHS pre-op vs 24 months’ follow-up values in subgroups of revision type.

Revision Type	*n*. of Patients	Total HHS Value			*p*
		Pre-op Mean	24 Months Follow-Up Mean	Variation Mean	
Partial revision	18	51.2	88.8	37.6 (CI: 28.7%–46.4%)	<0.001
Total revision	11	44.3	84.5	40.2 (CI: 26.6%–53.3%)	<0.001

**Table 8:** Total HHS pre-op vs 24 months’ follow-up values in subgroups of hip revision number.

Hip Revision Number	*n*. of Patients	Total HHS Value			*p*
		Pre-op Mean	24 Months Follow-Up Mean	Variation Mean	
First revision	17	50.5	91.9	42.4 (CI: 34.0%–50.7%)	<0.001
Second revision	7	48.7	84.0	35.3 (C.I. 14.2%–56.4%)	0.006
Third revision	5	45.4	75.6	30.2 (C.I. 5.8%–54.6%)	0.026

**Table 9:** Total HHS pre-op vs 24 months’ follow-up values in subgroups of cup type.

Cup Type	*n*. of Patients	Total HHS Value			*p*
		Pre-op Mean	24 Months Follow-Up Mean	Variation Mean	
Sansone iliac screw cup	6	46.2	74.0	27.8 (CI: 10.2%–45.4%)	0.01
Press-fit cup without iliac screw	23	49.3	90.6	41.3 (C.I. 33.5%–49.2%)	0.001

**Table 10:** Axial plane changes comparing 5–10 weeks with 12-month follow-ups in identified subgroups.

Cup Type	*n*. of Patients	Axial Plane (Mean) 1st CT	Axial Plane (Mean) 2nd CT	Decrease of Means (%)	*p*
Sansone iliac screw cup	5	309	252.4	−56.6 (20.2%)	0.009
Press-fit cup without iliac screw	9	342.9	256.7	−86.2 (25.6%)	<0.001

**Table 11:** Coronal plane changes comparing 5–10 weeks with 12-month follow-ups in identified subgroups.

Cup Type	*n*. of Patients	Coronal Plane (Mean) 1st CT	Coronal Plane (Mean) 2nd CT	Decrease of Means (%)	*p*
Sansone iliac screw cup	5	325.1	271.3	−53.8 (18.3%)	0.02
Press-fit cup without iliac screw	9	315.6	243.6	−72 (22.9%)	0.002

## Data Availability

The data presented in this study are available on request from the corresponding authors.

## Supplementary Materials

The following supporting information can be downloaded at: https://www.mdpi.com/article/10.3390/jcm11216372/s1, Figure S1: Examples of patients with a type III defect who were treated without the need for augments.
