# Supplementary material for: An Observational Prospective Clinical Study for the Evaluation of a Collagen-Hydroxyapatite Composite Scaffold in Hip Revision Surgery
Source: J Clin Med. 2022 Oct 28;11(21):6372. doi: 10.3390/jcm11216372 (PMC9654158; doi:10.3390/jcm11216372)
Supplement: Supplementary file 1 [file jcm-11-06372-s001.zip › jcm-1938922-SI.pdf]

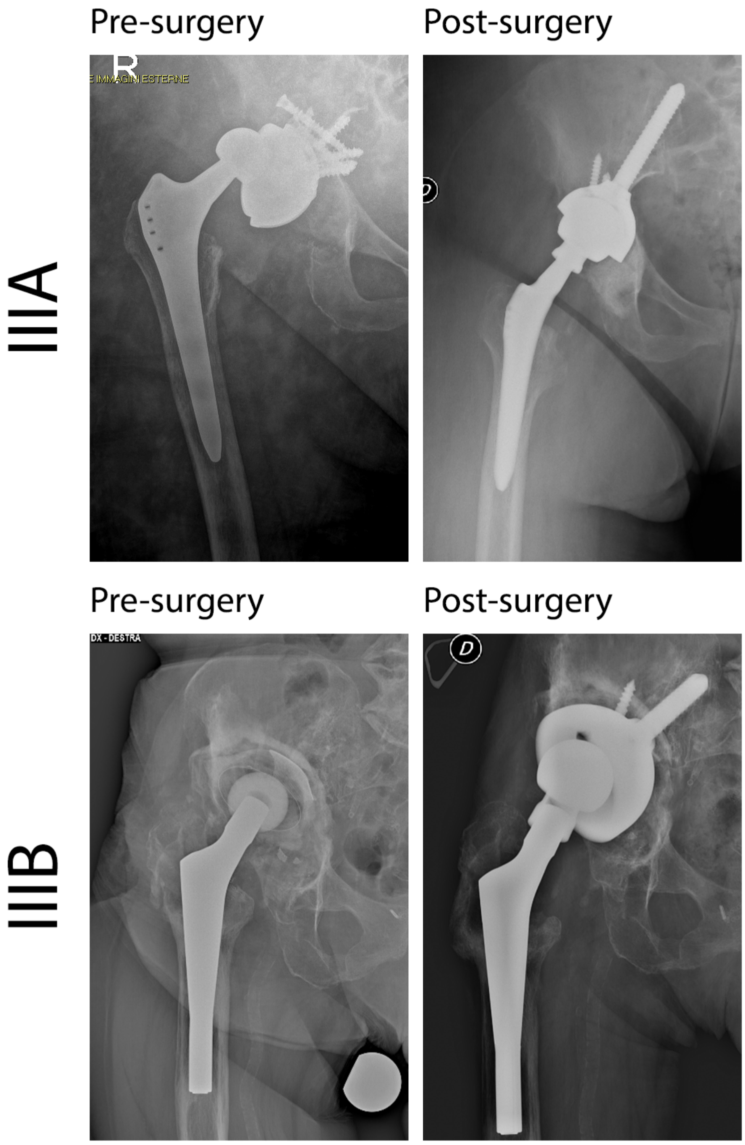

Figure S1. Examples of patients with a type III defect who were treated without the need for augments.
